# Biomolecular, Histological, Clinical, and Radiological Analyses of Dental Implant Bone Sites Prepared Using Magnetic Mallet Technology: A Pilot Study in Animals

**DOI:** 10.3390/ma14226945

**Published:** 2021-11-17

**Authors:** Gianmario Schierano, Domenico Baldi, Bruno Peirone, Mitzy Mauthe von Degerfeld, Roberto Navone, Alberto Bragoni, Jacopo Colombo, Riccardo Autelli, Giuliana Muzio

**Affiliations:** 1 Department of Surgical Sciences, C.I.R. Dental School, University of Turin, Via Nizza 230, 10126 Torino, Italy; 2 Department of Surgical Science (DISC), Division of Prosthetic Dentistry, University of Genoa, 16132 Genoa, Italy; baldi.domenico@libero.it (D.B.); jacopocolo@tiscali.it (J.C.); 3 Department of Veterinary Sciences, University of Turin, Largo Paolo Braccini 2, Grugliasco, 10095 Torino, Italy; bruno.peirone@unito.it (B.P.); mitzy.mauthe@unito.it (M.M.v.D.); 4 Department of Medical Science, University of Turin, Via Santena 5, 10126 Torino, Italy; navone.roberto@gmail.com (R.N.); alberto.bragoni@edu.unito.it (A.B.); 5 Department of Clinical and Biological Sciences, University of Turin, Corso Raffaello 30, 10125 Torino, Italy; riccardo.autelli@unito.it (R.A.); giuliana.muzio@unito.it (G.M.)

**Keywords:** dental implants, mallet technique, drill technique, implant stability quotient, osteogenesis, inflammation

## Abstract

Background. A new instrumentation exploiting magneto-dynamic technology (mallet) proposed for implant site preparation was investigated. Methods. In the tibias of three minipigs, two sites were prepared by mallet and two by drill technique. Primary stability (ISQ) was detected after implant positioning (T0) and at 14 days (T14). X-rays and computed tomography were performed. At T14, bone samples were utilized for histological and biomolecular analyses. Results. In mallet sites, histological evaluations evidenced a significant increase in the newly formed bone, osteoblast number, and a smaller quantity of fibrous tissue. These results agree with the significant BMP-4 augmentation and the positive trend in other osteogenic factors (biological and radiological investigations). Major, albeit IL-10-controlled, inflammation was present. For both techniques, at T14 a significant ISQ increase was evidenced, but no significant difference was observed at T0 and T14 between the mallet and drill techniques. In mallet sites, lateral bone condensation was observed on computed tomography. Conclusions. Using biological, histological, clinical, and radiological analyses, this study first shows that the mallet technique is effective for implant site preparation. Based on its ability to cause osseocondensation and improve newly formed bone, mallet technology should be chosen in all clinical cases of poor bone quality.

## 1. Introduction

Current bone surgery techniques aim to be less invasive and to accelerate healing processes.

New instruments have been designed for implant bone site preparation, as an alternative to drills, to reduce surgical trauma and phlogosis, obtain greater control of the cut, increase primary and secondary stability, and reduce healing times and morbidity [1,2,3,4,5].

Healing times and the osseointegration process depend on the cascade of biological events, including inflammation and osteogenesis. Therefore, it is necessary to analyze the biological factors involved, such as pro-and anti-inflammatory cytokines and bone morphogenic proteins (BMPs), molecules responsible for osteoblast/osteoclast differentiation or interactions between cells and the extracellular matrix [6,7]. Studies evaluating the biological mechanisms induced by the different preparation techniques and leading to the osseointegration of dental implants are very few. Recently, a new instrumentation exploiting magneto-dynamic technology has been proposed for bone surgery, including dental implant site preparation [8,9,10,11,12,13,14,15,16,17]. The literature on this technique is very limited and includes only observational clinical studies regarding the osseocondensation compared to conventional implant site preparation using drill technique. No reports have investigated the histological or biomolecular aspects.

Based on the above observations, this study aimed to compare implant bone site preparation using magneto-dynamic technology with that using the drill technique to identify the most effective means of improving implant osseointegration.

## 2. Materials and Methods

### 2.1. Animals

Three male adult minipigs, weighing 70–85 kg, older than two years, and showing the same characteristics of age, sex, weight, and health status were included in this study. They were drawn numerically from a lot of 15 minipigs by a dedicated veterinarian, not involved in the study, responsible for animal logistics. Animals were housed in single boxes in a dedicated room with controlled temperature and humidity at the Animal Pathology Department of Turin University, Italy. Minipigs received a standard pelleted cereal diet and water ad libitum.

The research was performed as a blinded study and in accordance with the Declaration of Helsinki. The study was approved by the Italian Ministry of Health (General Management for Animal Health and Veterinary Drugs. Office 6, Authorization number 304/2020-PR on 14 April 2020) and was conducted in compliance with the ARRIVE guidelines [18].

### 2.2. Focal Point

About the PICO (patient, intervention, comparison, and outcome), this study aimed to answer to the question “Does the magneto-dynamic mallet technique for implant site preparation, compared to conventional drilling technique, improve implant osseointegration?”.

○ Population: three minipig animals (we performed biomolecular and histological analyses on 3 implants for each technique).
○ Intervention: magneto-dynamic mallet technique for implant site preparation.
○ Comparison: conventional drilling for implant site preparation.
○ Outcomes: improving implant osseointegration.

### 2.3. Implant Insertion and Explantation

Before surgery, food and water were withheld for 12 h. Animals were given intramuscular meloxicam (5 mg/kg) (Inflacam 20 mg/mL, Laboratoires Virbac, Carros, France) 12 h before surgery. After induction with intramuscular xylazine (2.2 mg/kg) (Rompun 20 mg/mL, Bayer S.p.a., Milan, Italy) and tiletamine/zolazepam (6.6 mg/kg) (Zoletil 200 mg/mL, Laboratoires Virbac, Carros, France), an oro-tracheal tube was positioned and anesthesia was maintained with oxygen/isoflurane. Heart and respiratory rate, end-tidal CO_2_ (EtCO_2_), and oxygen saturation (SpO_2_) were monitored. In addition to preemptive meloxicam administration, minipigs received butorphanol (0.03 mg/kg IV) (Dolorex 1 mg/mL, Intervet, Aprilia, Italy) prior to surgery.

A conventional X-ray of the tibia of the left hind leg was carried out before surgery to examine the tibial anatomy. Each tibia was set up in a sterile way and exposed by ungluing the periosteum. Twelve titanium implants (ProActive Tapered 9 mm × 4 mm, implant neck 4.3 mm. Neoss^®^, Implants, Milano, Italy) were surgically inserted into the tibias. For each animal, the implant sites were prepared using the magnetic-dynamic mallet technique (Meta Ergonomica, Turbigo, Milano, Italy) (two implants) or the drill technique (two implants) according to the Neoss^®^ protocol (Neoss^®^, Implants, Milano, Italy).

All implant sites were prepared, at the cortical level, at 4 mm in diameter, applying the specific protocols for each type of instrumentation, allowing the same conditions for each single site.

For the mallet technique, the following inserts were used: PF 10–160 F–200 F–230 F, program Power 2 to 4. For the drill technique: lanceolate drill–2.2 mm Ø–3 mm Ø–3.2 mm Ø–3.4 mm Ø–3.6 mm Ø–countersink 4 mm Ø. Drills were used at 1300 rpm and countersink at 350 rpm.

For the first animal, the first two implant bone sites were randomly selected; in the other animals, the sites were chosen so that each type of preparation was situated in each tibia position (proximal, central, caudal) to avoid giving an advantage to one or the other technique.

Intraoperatively, the cortex bone thickness, using an appropriate instrument, and the bone quality were evaluated. All the implants were placed with a torque ranging from 35 to 70 (N·m); when necessary, the wrench was used to position the implant neck at the bone cortical level.

A distance of 5–6 mm was maintained between the two sites prepared with the same technique; the distance between the different preparations was greater than 9 mm. This distance was chosen to avoid overlap of biological phenomena between the two techniques.

The primary stability at four points (proximal, medial, lateral, and caudal) using the Penguin RFA instrument (Neoss^®^, Penguin, Milano, Italy) was detected for a total of 12 measurements for each implant. A bone sample for evaluating the biomolecular basal conditions was taken. The cover screws were placed, and a layered closure of the tissues was performed (T0). A second X-ray was taken to verify that the implants did not engage to the opposite cortex.

After surgery, the animals were treated for three days with meloxicam (5 mg/kg) (Inflacam 20 mg/mL, Laboratoires Virbac, Carros, France) as painkiller, and for five days with ceftiofur (3 mg/kg) (Norbrook^®^ Laboratories, Newry, Northern Ireland) as antibiotic therapy. At 14 days (T14), after anesthesia with intramuscular xylazine (2.2 mg/kg) (2.2 mg/kg) (Rompun 20 mg/mL, Bayer S.p.a., Milan, Italy) and tiletamine/zolazepam (6.6 mg/kg) (Zoletil 200 mg/mL, Laboratoires Virbac, Carros, France), the tibia of each animal was subjected to a computerized tomographic (CT) scan (Siemens Somatom Emotion Computerized Tomograph 16, Siemens Healthineers, Milano, Italy) with the following parameters: 160 mA, 130 KV, and 1 mm thickness. CT scans, in helical acquisition mode, were acquired with the subject in lateral decubitus with the limb under examination placed dorsally and in the center of the gantry, to repeat the same position for each animal. Each CT image was reconstructed in 3D and evaluated by two blinded operators in the advanced imaging diagnostics, not aware of either the implant sites or the surgical procedures.

All investigations were repeated three times at multiple different points.

Subsequently, the tibias were exposed as previously described; on each implant, the primary stability using the Penguin RFA (Neoss^®^, Penguin, Milano, Italy) instrument was detected.

**Figure 1:**
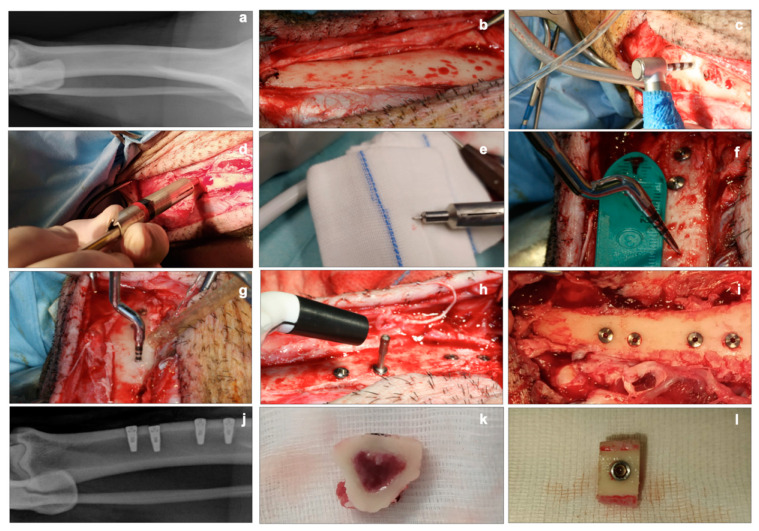
Clinical aspects. (**a**): conventional X-ray of the tibia before surgery. (**b**): exposure of the tibial bone after ungluing of the periosteum. (**c**): implant site preparation using a drill. (**d**,**e**): device used for intraoperative measurement of cortical thickness. (**f**,**g**): distance measurement for implant site preparation, using the mallet technique. (**h**): detection of the implant stability quotient (ISQ) with the Penguin RFA instrument at T0. (**i**): clinical view of the four implants inserted (two sites with the mallet technique, two sites with drill technique). (**j**): conventional X-ray of the tibia after implants placement. (**k**,**l**): bone slices for histological and biomolecular analyses.

For the two procedures in each animal, two bone slices (one mallet and one drill) were utilized for histology and the other two for biomolecular analyses. The final euthanasia was performed by an intracardiac injection of embutramide, mebezonium iodide, and tetracaine hydrochloride (70 mg/kg) (Tanax, Intervent International GmbH, Unterschleißheim, Germany) (Figure 1).

### 2.4. Histological Analyses

After implant removal, bone specimens were immediately fixed in 10% neutral buffered formalin and maintained at room temperature. Then, they were decalcified for at least 72 h in a mixture of formic and hydrochloric acids (BIODEC R, Bio Optica Milano, Italy), sectioned along the longitudinal implant axis and embedded in paraffin. From each bone portion, sections (5 µm) were obtained and stained with hematoxylin-eosin (H&E) for optical microscopy. The following parameters were analyzed in peri-implant tissue corresponding to 2 mm around each implant site: (1) maximum length of the newly formed bone (mm) measured from the implant profile; (2) bone tissue percentage; (3) number of osteoblasts. All data represent the mean of 10 fields.

Furthermore, sections stained with H&E were digitized using the Hamamatsu’s Nanozoomer 2 scanner (Aperio ImageScope, Buccinasco, Milano, Italy). Areas of new bone deposition and fibrous tissue were marked using an imaging computer software (Aperio ImageScope, Buccinasco, Milano, Italy), analyzed according to a protocol proposed by Han, J.-M. et al. [19], and expressed as total surface area.

### 2.5. Biomolecular Analyses

To protect mRNA, the specimens were placed in RNALater (Thermo Fisher Scientific, Monza, Italy) immediately after removal from the animals and stored at −80 °C until use. To perform the biomolecular analyses on the bone closest to the implant and actual site of osseointegration, specimens were cut so that the volume used for mRNA extraction was similar and corresponded to 2 mm around each implant site.

To obtain bone powder, samples were ground under a liquid nitrogen stream using a surgical stainless steel mortar and pestle. Total RNA was extracted from the powder (150 to 200 mg) using TRI Reagent (Merck Life Science, Milano, Italy).

For each sample, 1 μg of total RNA was reverse transcribed to cDNA using the FIREScript RT cDNA synthesis Kit (Solis Biodyne, Tartu, Estonia). Real-time PCR was performed on cDNA using 5 x HOT FIREPol^®^ Evagreen^®^ qPCR Supermix (Solis Biodyne, Tartu, Estonia). The forward (FW) and reverse (RV) primers, designed using the Primer3 tool available at https://bioinfo.ut.ee/primer3, accessed on 26 January 2021, are reported in Table S1 from Supplementary Material.

The expression of the following genes was evaluated at the bone-implant interface:

1. Osteogenesis: BMP-4; BMP-7; transforming growth factor-beta2 (TGF-β2); RUNX2, alkaline phosphatase (ALP); osteocalcin (OCN); collagen type I α1 (COLL1A1); Wnt3a; Wnt5a; Wnt10b; Wnt16.
2. Inflammation: interleukins (IL-1β; IL-6; IL-8; IL-10), tumor necrosis factor alpha (TNF-α).

Each sample was tested in triplicate and the quantitation cycle (Cq) values averaged. GAPDH was used as the housekeeping gene. The relative changes in the expression of different targets were defined as relative expression compared to that present in the corresponding bone sample T0, calculated as 2^−∆∆Cq^, where ∆Cq = Cq_sample_ − Cq_housekeeping_ and ∆∆Cq = ∆Cq_sample T14_ − ∆Cq_sample T0_.

### 2.6. Statistical Analysis

All data are expressed as mean ± SEM. Differences between group means were assessed using the Instat package, Version 3.10 (GraphPad Software, San Diego, CA, USA). Data were analyzed by unpaired two-tailed Student’s *t*-test or one-way ANOVA analysis followed by the Bonferroni post hoc test. A *p*-value < 0.05 was considered statistically significant. Pearson’s correlation coefficient (*r*) was computed to investigate the linear dependence of the ISQ and the cortical bone thickness.

## 3. Results

### 3.1. Biomolecular Data

**Figure 2:**
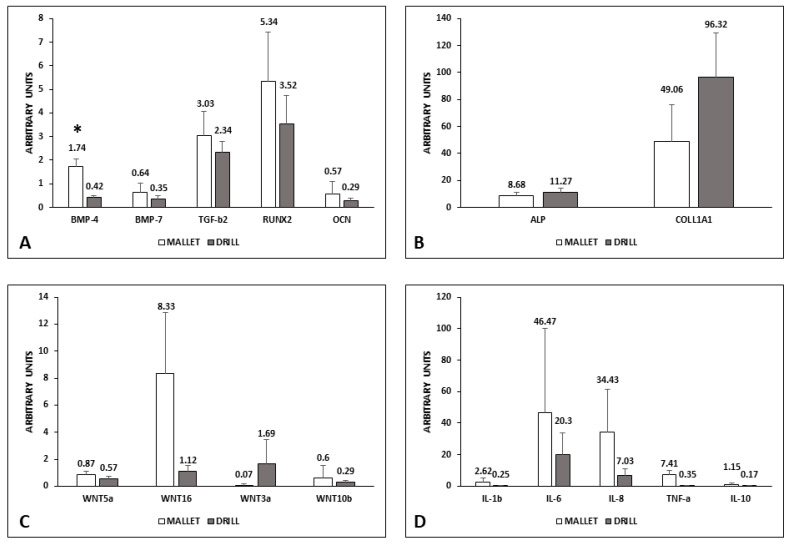
mRNA level of osteogenic and inflammatory factors in peri-implant bone tissues. Panels (**A**,**B**): osteogenic factors; panel (**C**): WNT pathways; panel (**D**): inflammatory factors. Data represent the mean ± SEM of the evaluations carried out on bone samples obtained at T14. For each animal, the mRNA amounts detected at this time were normalized with respect to the values found in the corresponding bone at T0. Student’s *t* Test: BMP-4 * *p* = 0.017. BMP-4, bone morphogenetic protein-4; BMP-7, bone morphogenetic protein-7; TGF-β2, transforming growth factor-β2; RUNX2, RUNX family transcription factor 2; OCN, osteocalcin; ALP, alkaline phosphatase; COLL1A1, collagen 1A1; WNT, wingless-related MMTV integration site; IL-1β, interleukin-1β; IL-6, interleukin-6; IL-8, interleukin-8; TNF-α, tumor necrosis factor-α; IL-10, interleukin-10.

The mRNA content of the factors involved in the induction of bone synthesis is reported in Figure 2.

In tissues surrounding implants using the mallet technique, the expression of BMP-4, BMP-7, TGF-β2, RUNX2, and OCN was higher than those with drill ones. Only the difference in BMP-4 was statistically significant (Panel A). Conversely, ALP and COLL1A1 were lower in mallet sites (Panel B).

The canonical Wnt3a was less expressed in the case of mallet instrumentation, whereas all the other members examined (Wnt5a, Wnt10b, and Wnt16) were higher than in drill sites (Panel C). Moreover, regarding Wnt expression, the differences were not statistically significant.

Pro-inflammatory cytokines IL-1β, IL-6, Il-8, and TNFα showed no significant increase in mallet sites; in these sites, a similar trend was observed in anti-inflammatory IL-10 (Panel D).

### 3.2. Histological Data

**Figure 3:**
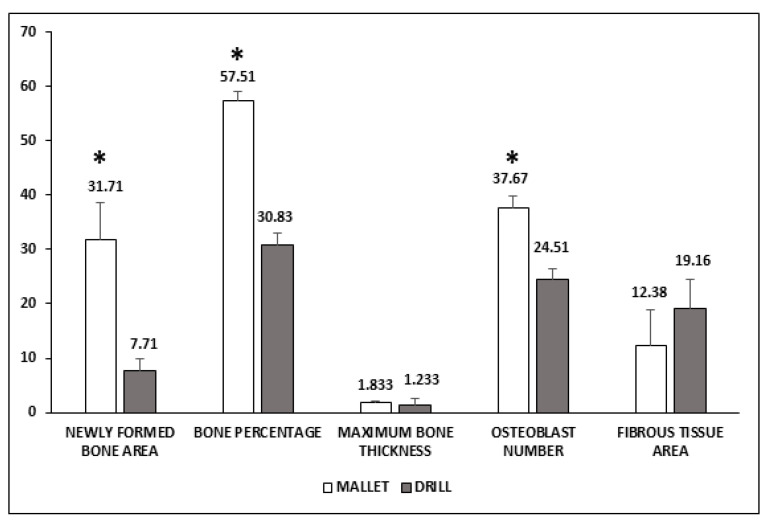
Histological evaluation of peri-implant bone tissues. Areas of newly formed bone and fibrous tissues are expressed as mm^2^ and have been evaluated using an imaging computer software. Maximum bone thickness is expressed as mm and was evaluated, as the osteoblast number, by optical microscopy. Data represent the mean ± SEM of the evaluations carried out on bone samples obtained at T14. Student’s *t*-test: Newly Formed Bone Area * *p* = 0.031; Bone Percentage * *p* = 0.001; Osteoblast number * *p* = 0.009.

Figure 3 shows that in mallet sites, a significant increase of newly formed bone area, bone percentage, and osteoblast number was present. The greater amount of bone tissue was coupled with a smaller, nonsignificant amount of fibrous tissue**.** No significant increase in the maximum length of the newly formed bone was observed.

**Figure 4:**
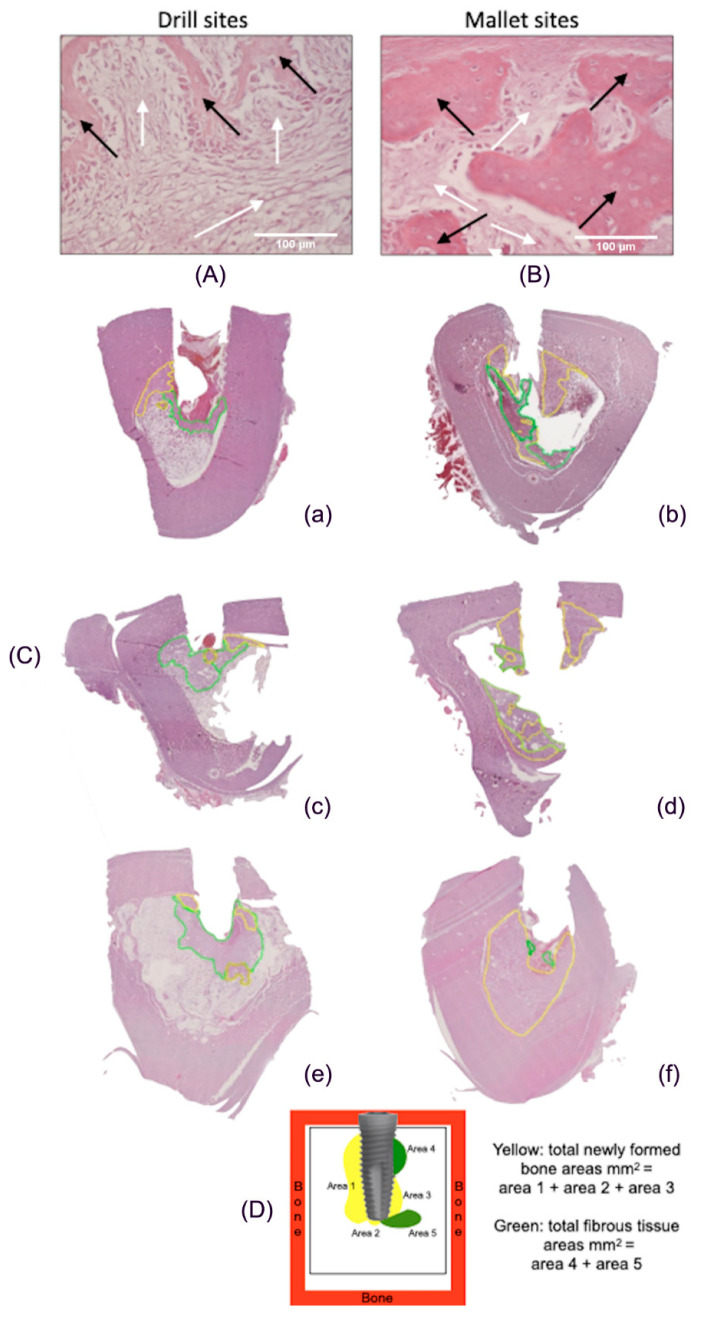
Histological images of peri-implant tissues and scheme of area calculation. (**A**) The fibrous tissue (white arrows) was greater than newly formed bone tissue (black arrows) in drill-prepared sites (Hematoxylin and Eosin, 400×). (**B**) In the mallet site, the amount of newly formed bone tissue (black arrows) is greater than that of fibrous tissue (white arrows) (Hematoxylin and Eosin, 400×). (**C**) Areas of new bone and fibrous tissue deposition, expressed as total surface (mm^2^), using an imaging computer software, according to the Han, J.-M. et al. [19] protocol. In every picture, in yellow, areas of new bone deposition and in green, areas of fibrous tissue. (**a**,**c**,**e**): tissues from sites prepared with drill technique, in the three different animals; (**b**,**d**,**f**): tissues from sites prepared with mallet technique in the three different animals. The bone samples obtained from mallet sites show a greater area of newly formed bone and a lower area of fibrous tissue compared to drill ones. (**D**) Example of scheme for calculating the newly formed bone and fibrous tissue around the implants.

Figure 4A,B reports histological pictures representative of newly formed bone (yellow line) and fibrous tissue (green line). In Panel C, representative images of the total peri-implant surfaces, measured by using computer imaging software, are illustrated. Panel D describes the scheme of the calculation of the tissue areas surrounding implant.

### 3.3. Clinical and Radiological Data

At all sites, the cortex showed type 1 quality, while the cancellous bone showed type 4 [20]. In mallet sites, the mean cortical thickness (mm) was 3.95 ± 0.30; in drill sites, 3.72 ± 0.316 (*p* = 0.51). The insertion torque was high in the sites prepared with the mallet technique: in one case, it was 50 (N·m), in all others 70, and in one of the latter cases, a wrench was needed. At the drill sites, the insertion torque ranged from 35 to 59 (N·m); in two cases, it reached 70. For both techniques, a significant increase in ISQ was evidenced at T14 compared with the corresponding T0; conversely, no significant difference was observed at T0 and T14 between the mallet and drill ISQ values. No correlation was evidenced between cortical thickness and ISQ values (Table 1).

**Figure 5:**
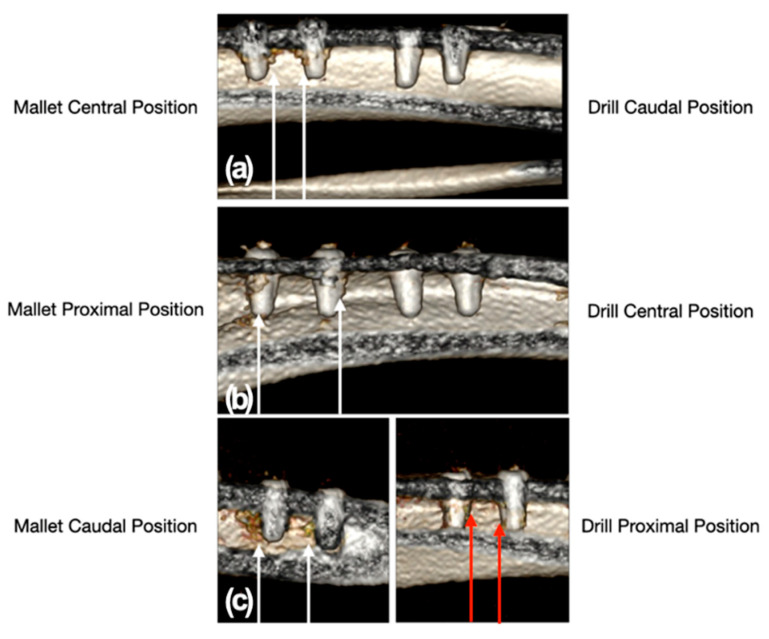
CT scan images of the position of the implant sites relative to the tibial bone. Panel (**a**): mallet in central and drill in caudal position; panel (**b**): mallet in proximal and drill in central position; panel (**c**): mallet in caudal and drill in proximal position. The white arrows indicate the two sites, with the relative implants, prepared with the mallet technique. In these sites, a moderate trabecular densification organized at the cortico-cancellous junction adjacent to all implants could be noted. The sites prepared with drill showed no trabecular bone densification adjacent to the implants, except in one site where a slight, nonorganized trabecular densification with the presence of radiodense millimeter spots was present (red arrows).

The CT scan showed a moderate trabecular densification organized at the cortico-cancellous junction adjacent to all implants in the sites prepared with the mallet technique. By contrast, drill sites did not show trabecular bone densification adjacent to the implants, except in one site where a slight, nonorganized trabecular densification with the presence of radiodense millimeter spots was observed (Figure 5). The mean weight of explanted bone samples was 3.15 ± 0.20 g in mallet sites and 2.63 ± 0.37 g in the drill ones (*p* = 0.065).

## 4. Discussion

The magnetic-dynamic technique has recently been introduced in oral bone surgery, such as dental extraction, split crest, sinus lift, and implant site preparation. Only few observational clinical studies, almost all conducted by the same group, investigated the efficacy of this new instrumentation [8,9,10,11,12,13,14,15,16,17]. To the best of our knowledge, this is the first study exploring, by means of clinical, radiological, histological, and biological analyses, the effects of mallet instrumentation on bone implant site preparation, compared with the drill technique. The minipig model was chosen because it is frequently used in dental implant research [2,7]. The implants were positioned in the tibia due to the difficulty inserting them in the oral cavity [21]. The analyses were performed at 14 days based on literature showing that significant changes in primary implant stability and osseointegration process are already present at this time [2,22].

### 4.1. Biological Factors

#### 4.1.1. Osteogenic Process

The expression of genes involved in early and late stages of osteogenesis was evaluated.

BMPs are cytokines belonging to the TGF-β superfamily which regulate several physiological processes. BMP-4 and BMP-7 possess strong osteogenic properties. BMP-4 is mainly responsible for recruitment of mesenchymal stem cells (MSC) and their commitment to the osteoblast lineage, inducing the transcription of osteoblast characterizing genes, such as *ALP, Osterix*, and *RUNX2* [23,24]. In rat calvaria osteoblasts, BMP-2-induced BMP-4 expression has been demonstrated to increase ALP, type I collagen, and OCN [25].

With a partially overlapping mechanism, BMP-7 induces the expression of ALP and OCN and favors bone mineralization [26,27,28,29]. Among the BMP-induced osteogenic factors, OCN plays a crucial role in the last phase of bone deposition, contributing to matrix mineralization [30]. The TGF-β pathway is involved in osteogenesis by stimulating the proliferation of osteoblast precursors and in the early phase of differentiation [31]. In our model, the expression of BMP-4, BMP-7, and TGF-β2 is increased in mallet sites compared with drill ones, even though the difference was only statistically significant for BMP-4. The increased expression of BMP-4 and 7 is associated with that of RUNX2 and OCN, genes known to be under transcriptional control of these BMPs.

To explain why ALP and COLL1A1 expression is lower in mallet sites, it could be suggested that in these sites the induction of osteogenesis occurs earlier, and the major expression of these factors could have occurred before 14 days. Regarding the lower COLL1A1 level, the hypothesis agrees with histological data showing a major bone tissue and a minor fibrous tissue. In sites prepared with the mallet technique, the increased expression of all osteogenic factors investigated (BMP-4, BMP-7, TGF-β2, OCN, and RUNX2) might be at the origin of the significant increase in newly formed bone area and number of osteoblasts, and of the nonsignificant increase in bone thickness, highlighted by the histological analysis.

#### 4.1.2. Inflammatory Process

The occurrence and the entity of the osteogenesis process are strictly correlated with the inflammatory response consequent to surgery injury. It is well known that some inflammatory mediators also favor tissue regeneration/repair. The determination of pro-inflammatory molecules revealed that in mallet sites, the mRNA levels of IL-1β, IL-6, IL-8, and TNF-α were higher than in drill ones, although in this case the differences were also not significant.

However, in mallet sites, the inflammation seemed to be under control; in fact, the bone deposition and the number of osteoblasts were greater in these sites than in those prepared with a drill. In mallet sites, the action of pro-inflammatory cytokines was probably reduced by the increased expression of IL-10 and TGF-β2, which negatively modulate phlogosis [32].

#### 4.1.3. Wnt

Wnt pathway is involved in regulation of signal transduction from outside to inside the cell, cell proliferation, differentiation, polarity, and inflammation [33,34,35,36,37]. It can be divided into: 1. canonical, which causes the stabilization of β-catenin; 2. noncanonical, which works independently of β-catenin.

Both pathways concur in the osseointegration of dental implants and are upregulated on porous titanium implant surfaces [38]. It has been reported that a high level of Wnt signaling at the moment of implant positioning prevents excessive deposition of fibrous tissue and favors osseointegration [39]. It has been shown that Wnt/β-catenin promotes the expression of Runx2, ALP, BMP-7 [40], and noncanonical pathway up-regulates BMP-2 and BMP-4 [41]. Olivares-Navarrete et al. evidenced that, during osseointegration on microstructured and hydrophilic surface of grade 2 unalloyed Ti, the canonical pathway is activated in early-stage of differentiation of osteoblast-like MG63 cells, and noncanonical in late-stage of differentiation, improving osseointegration [42].

In our study, we investigated the expression of some canonical (Wnt3a, 10b) and noncanonical (Wnt5a, 16) proteins. In the sites prepared using the mallet technique, Wnt3a was less expressed than in drill sites; by contrast, Wnt5a was increased. These results agree with previous reports showing that the transient exposure to Wnt3a causes a quick and early bone formation and that Wnt5a inhibits Wnt3a [43].

The major amount of bone in the mallet sites could reflect a more advanced phase of osteogenesis due to the Wnt5a increase at 14 days. The low levels of Wnt3a in the mallet sites, at 14 days, may explain the low levels of ALP and COLL1A1 since these genes are under the transcriptional control of Wnt3a and normally increase early in bone healing. The major quantity of bone observed in histological analysis in tissues surrounding mallet sites could also be due to the increased expression of Wnt16 and Wnt10b. The former blocks osteoclast differentiation [44]; the latter triggers transcription of genes that drive MSC osteogenetic differentiation [45].

The osteogenic property of Wnt10b has been indirectly confirmed by the observations that mice that do not express it, show a reduction of bone mass and that, in mouse embryonal development, the expression of Wnt10b induces fibroblast differentiation to osteoblasts [46]. The increased Wnt5a can also explain the increment of the inflammatory indices, since it has been reported that IL-1β, IL-6, IL-8, and TNF-α increase consequent to Wnt5a enhancement [47,48].

### 4.2. Histological Analyses

Histological analyses were used, together with the biomolecular analyses, to outline a complete view of osseointegration process. The results obtained with the optical microscope and computerized analysis showed a significant increase in the deposition of newly formed bone and in the number of osteoblasts, associated with a lower quantity of fibrous tissue, in the sites prepared using the mallet technique. It is to be noted that newly formed bone probably also includes the “scar” fibrous tissue and bone spicules formed by the osseocondensation induced by the surgical mallet technique. These observations agree with the increase in osteogenic factors and the decrease of collagen found with biomolecular analyses, even though the differences are not statistically significant. The discrepancy in terms of statistical significance between the two types of investigations could be because the histological evaluations present a picture that is the result of previous changes while biological parameters are the basis for changes that will occur in the following healing times.

### 4.3. Clinical and Radiological Observations

Positioning of the implants in different portions of the tibia did not affect the results obtained from either surgical technique. The mean cortical thickness and mean weight of bone samples did not statistically differ between the two experimental groups. However, the mean cortical thickness of tibias influenced the preparation of bone implant sites using the mallet device. In fact, before its use, it was necessary to make small holes for almost the entire thickness of the cortex with a lanceolate drill.

In this way, the cortical portion was weakened to favor the progression of the subsequent magneto-dynamic inserts suitable for site preparation. After this action, the progression of the inserts occurred without problems. However, the fact that it was necessary to make small holes in the case of a very thick and dense cortex could suggest that the mallet technique shows a minor penetration capacity, probably due to the reduced instrument power or to the not very aggressive design of the first insert.

Furthermore, it can be suggested that the inserts be used starting from power 2 of the slider; only when the penetration capacity of the insert is reduced can the power be progressively increased reaching the maximum power of 4.

Starting the preparation using maximum power could cause rupture of cancellous bone trabeculae instead of pushing and compacting them sideways in the cancellous spaces. Moreover, even if mallet technology does not require irrigation, it is advisable to sometimes irrigate the site with sterile saline solution to preserve bone elasticity and reduce its rigidity, thus decreasing the occurrence of microfractures. Nevertheless, in our experimental conditions, the mallet device did not cause fractures; this is probably due not only to the intermittent irrigation, but also to its very rapid action in terms of impulse release [11]. These latter clinical-observational hypotheses require further investigations.

In our study, in the sites prepared with mallet technique, lateral bone condensation was observed in the presence of type 1 cortex and type 4 cancellous bone. This was confirmed by the blinded CT scan analysis. These observations agree with previous studies evidencing bone condensation in implant sites prepared with a magnetic mallet, using conventional X-rays [10,11,14] or CT [16,17]. Nevertheless, the increased stability detected from T0 to T14 with both techniques argues in favor of the effectiveness of the magneto-dynamic device, since the preparation with drills is morphologically dedicated to the implant used, while that with mallet instrumentation is not.

These results suggest that the increased stability in the case of the mallet technique is probably due to the cancellous osseocondensation caused by this instrument. This hypothesis seems to be confirmed by the increased implant insertion torque, the significantly higher amount of bone evidenced by histological analyses, and the clear trend to a greater expression of all osteogenic factors in mallet-compared to with drill-prepared sites.

### 4.4. Limitations of the Study

Although overall the results of this pilot study show, for the first time, that the mallet technique is effective for the preparation of the implant site, further studies are required to analyze the implant osseointegrative process at longer experimental times, with a larger sample size. Moreover, human trials are needed.

### 4.5. Value of the Study

This is the first study that investigates, by biomolecular, histological, radiological, and clinical analyses, implant site preparation using a magneto-dynamic technique.

### 4.6. Future Directions

Understanding the clinical impact of surgical instruments, in terms of use and performance, through biological studies should offer great benefits not only in dental implantology and oral or maxillofacial surgery but also in all fields relying on bone surgery.

## 5. Conclusions

The present study demonstrated that, in relation to the bone quality and the experimental time (14 days), the magnetic-dynamic mallet technique can significantly increase the amount of newly formed bone tissue and the quantity of osteoblasts compared with the drill technique, as shown by histological analyses. The intrinsic ability of the mallet to osteocondensate the bone tissue can positively affect the primary stability. In addition, an increase in osteogenetic biological parameters has been observed in these sites, apart from Wnt 3a, suggesting a positive trend regarding secondary implant stability. However, the mallet device was found to be less performing in terms of perforation in conditions of dense and thick bone quality, as cortical areas are. From these considerations, it can be affirmed that the magneto-dynamic turns out to be a technique of choice in the preparation of the maxillary implant site, in the case of poor bone quality or in all clinical conditions in which the cortex is thin or shows low quality.

## Figures and Tables

**Table 1:** Clinical data representing the values of the Implant Stability Quotient (ISQ), the cortex thickness, and their correlation.

**ISQ Values**			
**Mallet**		**Drill**	
**T0**	**T14**	**T0**	**T14**
77.531 ± 0.542 a	80.979 ± 0.441 b	76.250 ± 0.479 a	81.062 ± 0.455 b
**ISQ Percent Increase**			
**Mallet**		**Drill**	
4.592 ± 0.325		6.467 ± 0.525 *	
**Cortex Thickness (mm)**			
**Mallet**		**Drill**	
3.625 ± 0.311		3.475 ± 0.204	
**Correlation between Cortex Thickness and ISQ (r)**			
**Mallet**		**Drill**	
T0	T14	T0	T14
0.51	0.36	0.65	0.55

Data are expressed as mean ± S.E.M. Differences between group means were assessed using one-way ANOVA analysis followed by Bonferroni post hoc test or unpaired two-tailed Student’s *t-*test. Means with different letters are statistically different. A *p*-value < 0.05 was considered statistically significant. Student’s *t-*test: * *p* = 0.0028. Person’s correlation coefficient (*r*). ISQ, Implant Stability Quotient.

## Data Availability

Data sharing not applicable.

## Supplementary Materials

The following are available online at https://www.mdpi.com/article/10.3390/ma14226945/s1, Table S1. Primer Sequences for PCR Analysis.
