# Supplementary material for: Biomolecular, Histological, Clinical, and Radiological Analyses of Dental Implant Bone Sites Prepared Using Magnetic Mallet Technology: A Pilot Study in Animals
Source: Materials (Basel). 2021 Nov 17;14(22):6945. doi: 10.3390/ma14226945 (PMC8618607; doi:10.3390/ma14226945)
Supplement: Supplementary file 1 [file materials-14-06945-s001.zip › materials-1430363-supplementary.pdf]

materials

MDPI

Supplementary Material

Biomolecular, Histological, Clinical, and Radiological Analyses of Dental Implant Bone Sites Prepared Using Magnetic Mallet Technology: A Pilot Study in Animals

Gianmario Schierano [1,*], Domenico Baldi [2], Bruno Peirone [3], Mitzy Mauthe von Degerfeld [3], Roberto Navone [4], Alberto Bragoni [4], Jacopo Colombo [2], Riccardo Autelli [5] and Giuliana Muzio [5]

1 Department of Surgical Science, C.I.R Dental School, University of Turin, Via Nizza 230, 10126 Torino, Italy
2 Department of Surgical Science (DISC), Division of Prosthetic Dentistry, University of Genoa, 16132 Genoa, Italy; baldi.domenico@libero.it (D.B.); jacopocolo@tiscali.it (J.C.)
3 Department of Veterinary Sciences, University of Turin, Largo Paolo Braccini 2, Grugliasco, 10095 Torino, Italy; bruno.peirone@unito.it (B.P.); mitzy.mauthe@unito.it (M.M.v.D.)
4 Department of Medical Science, University of Turin, Via Santena 5, 10126 Torino, Italy; navone.roberto@gmail.com (R.N.); alberto.bragoni@edu.unito.it (A.B.)
5 Department of Clinical and Biological Sciences, University of Turin, Corso Raffaello 30, 10125 Torino, Italy; riccardo.autelli@unito.it (R.A.); giuliana.muzio@unito.it (G.M.)
* Correspondence: gianmario.schierano@unito.it; Tel.: +39-(0)11-6331531/1532; Fax: +39-(0)11-6331513

Table S1. Primer sequences for PCR analysis.

Gene	Accession Number	Primer Sequences
BMP-4	NM_001101031	FW 5′-ggcccctcctggtcacattt-3′<br>RV 5′-tccccatggcagtagaaggc-3′
TGF-β2	XM_021064293	FW 5′-gctgcctgcgtccactttac-3<br>RV 5′-cagagctccacaggtacggg-3′
RUNX2	XM_005666074	FW 5′-ccccatccatccactccacc-3′<br>RV 5′-aggcagaagtcagaggtggc-3′
ALP	AH012163.2	FW 5′-tcactgctgaccactcccac-3′<br>RV 5′-gggtttcttgtccgtgtcgc-3′
OCN	XM_013996978.2	FW 5′-gttccccaccttgtccacca-3′<br>RV 5′-ctcccagcccatgatccagg-3′
COLL1A1	AF201723	FW 5′-ggtgaacccggcaaacaagg-3′<br>RV 5′-gctccctcacgtccagactc-3′
BMP-7	NM_001105290	FW 5′-gaacagcagcagtgaccagc-3′<br>RV 5′-acgagttgagtgggaaggcg-3′
IL-1β	M86725	FW 5′-gcacactagggtcactggca-3′<br>RV 5′-gacactgctgcttcttggcc-3′
IL-6	JQ839263	FW 5′-gggaaatgtcgaggctgtgc-3′<br>RV 5′-ttgtggtggggttaggggtg-3′
TNFα	X57321	FW 5′-gagaatgaggggctggggac-3′

		RV 5′-tcttggtggtggtcagtgcc
IL-10	NM_214041	FW 5′-tctaccatgcccagctcagc-3′<br>RV 5′-caggctggttgggaagtgga-3′
IL-8	M86923.1	FW 5′-aggaccagagccaggaagaga3′<br>RV 5′-gccagaactgcagcctcaca-3′
WNT3a	XM_021083340	FW 5′-tgtgaacccaaccccgagac-3′<br>RV 5′-ctctcagtgcgtgcgttgtg-3′
WNT5a	XM_005669660.1	FW 5′-gctgcggagacaacatcgac-3′<br>RV 5′-gcaggttcatgaggatgcgc-3′
WNT10b	KF569219	FW 5′-gtgggaatggggtggctgta-3′<br>RV 5′-cgcattcgtgcctggatgtc-3′
WNT16	XM_003134736	FW 5′-cagcggcaccaaggaaacag-3′<br>RV 5′-ctgagccgccattctgcaag-3'
GAPDH	NM_001206359	FW 5′-ctctctgctcctccccgttc-3′<br>RV 5′-ggccaaatccgttcactccg-3′

All sequences used for designing primers are from Sus Scrofa. BMP-4, Bone Morphogenetic Protein-4; TGF-β2, Transforming Growth Factor-β2; RUNX2, RUNX Family Transcription Factor 2; ALP, Alkaline Phosphatase; OCN, Osteocalcin; COLL1A1, Collagen 1A1; BMP-7, Bone Morphogenetic Protein-7; IL-1β, Interleukin-1β; IL-6, Interleukin-6; TNFα,Tumor Necrosis Factor α; IL-10, Interleukin-10; IL-8, Interleukin-8; WNT, Wingless-related MMTV integration site; GAPDH, Glyceraldehyde 3-phosphate dehydrogenase.
